# Supplementary material for: Biodiversity-Driven Screening of Amphibian Skin Secretions for Inflammatory Modulation in Joint Diseases
Source: Toxins (Basel). 2025 Sep 17;17(9):464. doi: 10.3390/toxins17090464 (PMC12474187; doi:10.3390/toxins17090464)
Supplement: Supplementary file 1 [file toxins-17-00464-s001.zip › toxins-3808457-supplementary.pdf]

Supplementary Material

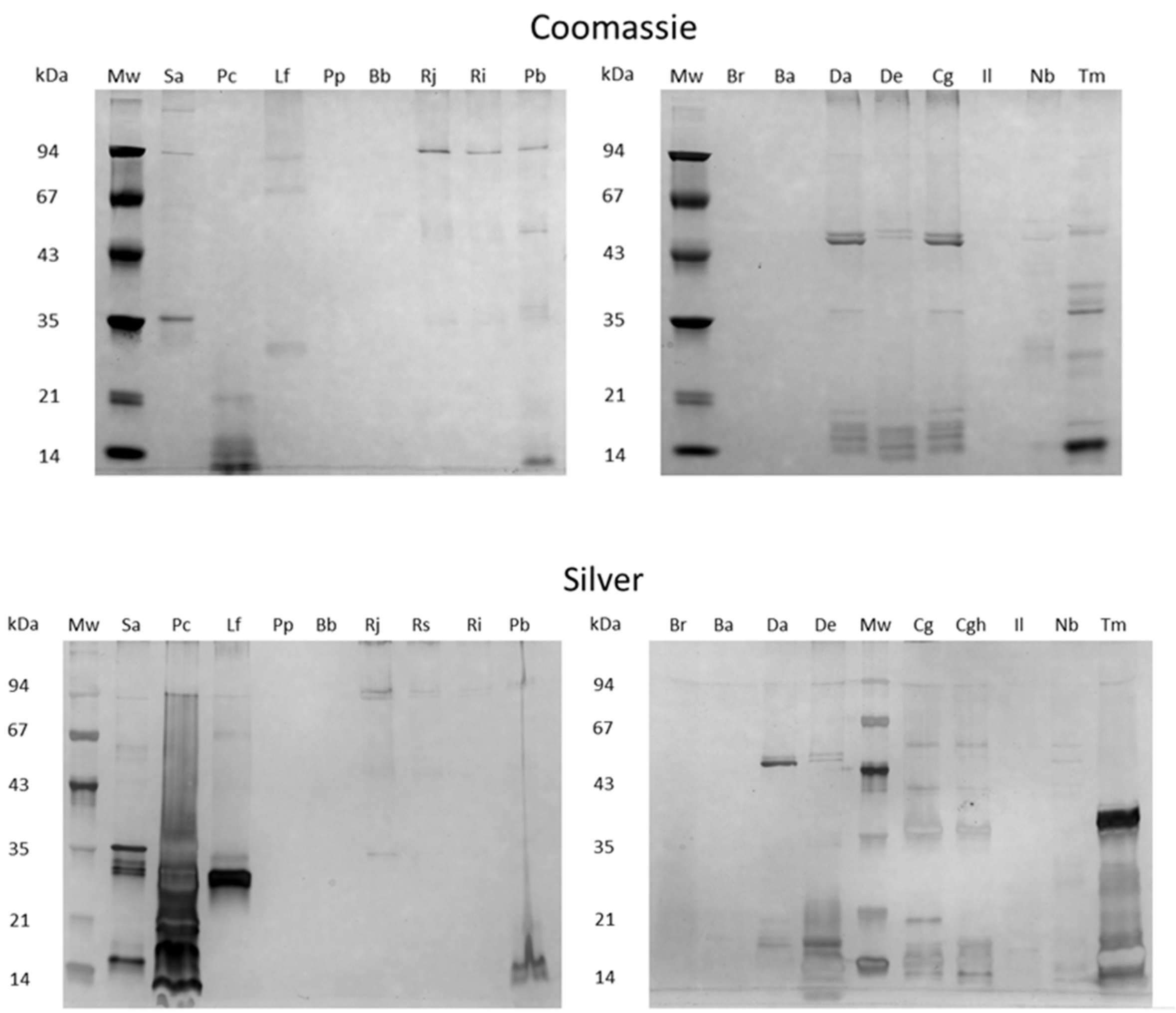

Figure S1. Original SDS-PAGE gels used in figure 1, Coomassie blue or silver stained as indicated. Secretions from *Siphonops annulatus* (Sa), *Pipa carvalhoi* (Pc), *Leptodactylus fuscus* (Lf), *Pristimantis paulodutrai* (Pp), *Bufo bufo* (Bb), *Rhinella jimi* (Rj), *R. schneideri* (Rs), *R. icterica* (Ri), *Phyllomedusa bahiana* (Pb), *Boana raniceps* (Br), *B. albomarginata* (Ba), *Dendropsophus anceps* (Da), *D. elegans* (De), *Corythomantis greeningi* (Cg body; Cgh head), *Itapotihyla langsdorfii* (Il), *Nyctimantis brunoi* (Nb) and *Trachycephalus mesophaeus* (Tm) (50 µL, 1 mg/mL) were run in 12% polyacrylamide gels. The gels were either silver or Coomassie blue stained, Molecular weight standard (MW) in kDa.

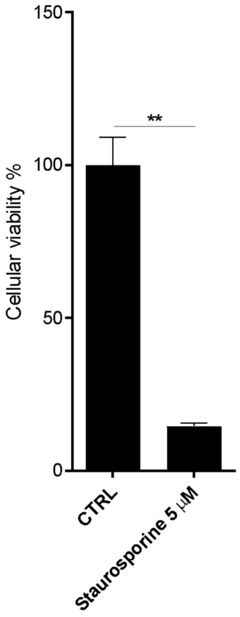

Figure S2. Effect of staurosporine on macrophage viability. Cellular viability was measured by MTT assay after 24 h of treatment with staurosporine at 5 µM. Data represent the mean ± SD. Significant differences between the control and treatments were evaluated using Mann-Whitney U test . (n = 3, ** $p < 0.01$; vs. control 100%).

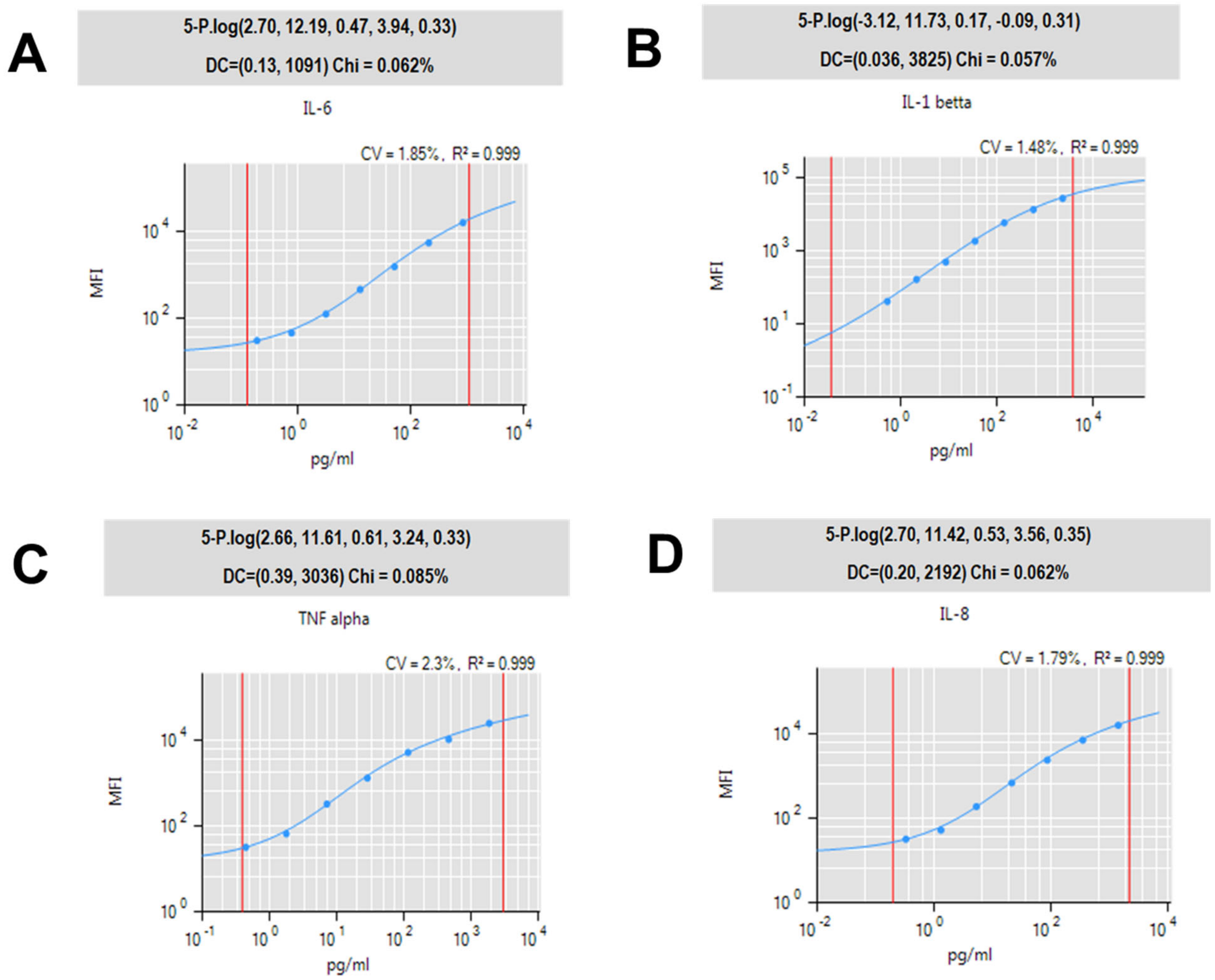

Figure S3. Representative standard curves generated by the Milliplex® multiplex cytokine assay report (#HCYTOMAG-60K). IL-6 (A), IL-1β (B), TNF-*α* (C) and IL-8 (D) curves were obtained using serial dilutions of recombinant cytokine standards provided in the kit, and fluorescence intensity (MFI) values were fitted to a five-parameter logistic (5-PL) regression model to calculate analyte concentrations. These standard curves served as the basis for the interpolation of cytokine concentrations in experimental samples.

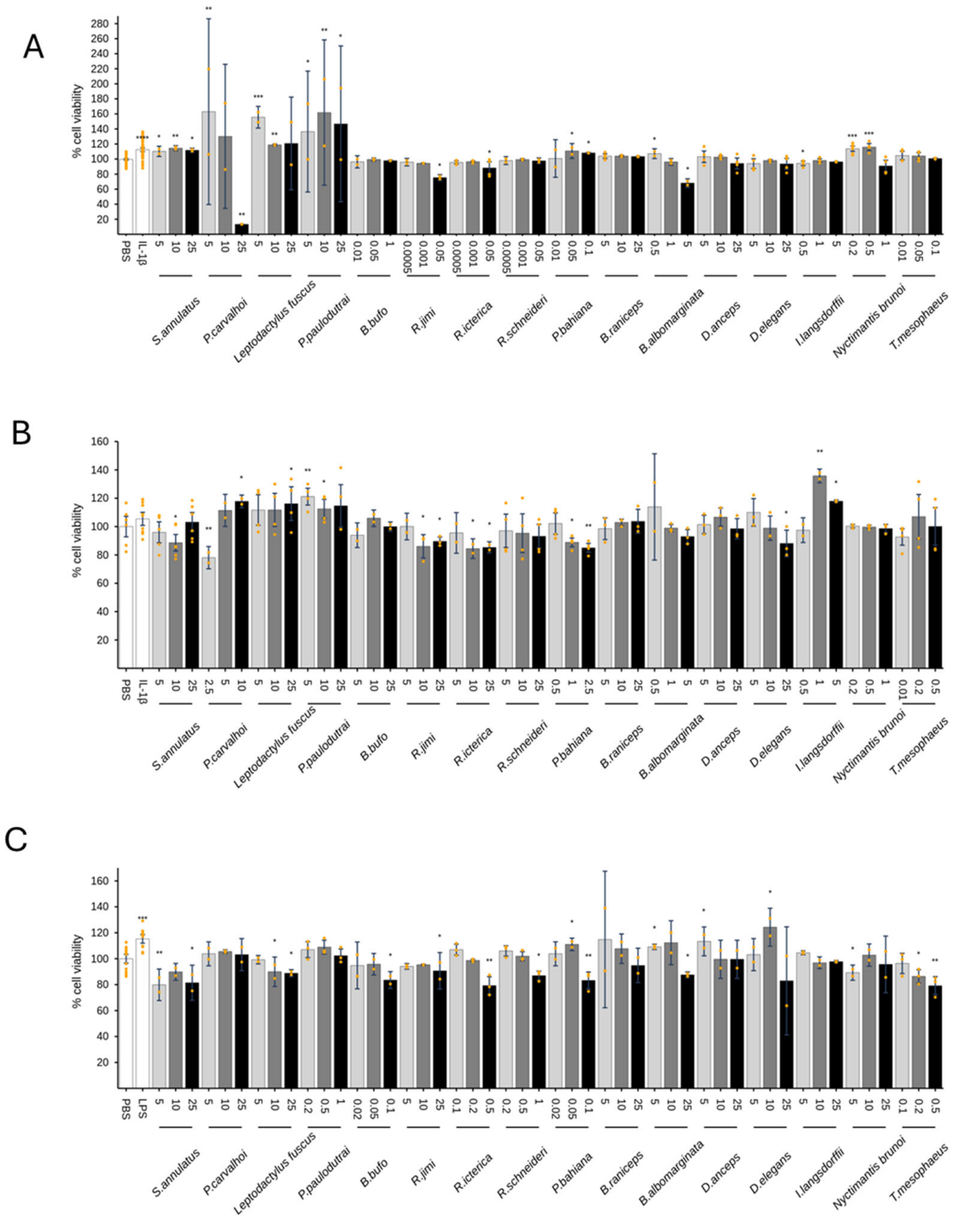

Figure S4. Determination of non-cytoxic concentrations of amphibian's secretion on chondrocytes (A), synoviocytes (B) and macrophages (C). Cells were treated with amphibian secretions at different concentrations for 24 h. The MTT assay measured cellular viability. Each data point is presented as the mean ± confidence interval error from two independent experiments. Statistical analysis was performed using Dunn's test without correction for multiple comparisons. The p-values are indicated as follows: * for 0.2> p > 0.05, ** for 0.05 > $p \geq$ 0.01, ** for 0.01 > $p \geq$ 0.001, and *** for p < 0.001.

Table S1. Amphibian species used in this study, with information on sex, size, collection locality, secretion extraction method, and voucher numbers deposited in the Amphibian Collection of the Laboratory of Structural Biology, Butantan Institute (LBC-A).

Species	Sex	Size	Collection locality	Extraction method	Voucher number
Boana albomarginata	un	adult	Ilhéus (BA)	bmcw	LBC-A 1105; LBC-A 1110
Boana raniceps	un	adult	Maraú (ES)	bmcw	LBC-A 1250
Bufo Bufo	not apply	not apply	*Acquired from market	not apply	not apply
Corythomantis greeningi	un	adult	Angicos (RN)	bmcw	LBC-A 1125; LBC-A 1031; LBC-A 1044
Dendropsophus anceps	un	adult	Igrapiúna (BA)	bmcw	LBC-A 1179; LBC-A 1197; LBC-A 1240
Dendropsophus elegans	un	adult	Igrapiúna (BA)	bmcw	LBC-A 1183; LBC-A 1160
Itapotihyla langsdorffii	un	adult	Santa Cruz (ES)	bmcw	LBC-A 1176
Leptodactylus sp	un	adult	Ilhéus (BA)	bmcw	LBC-A 1122; LBC-A 1251; LBC-A 1308
Nyctimantis brunoi	un	adult	Santa Cruz (ES)	bmcw	LBC-A 1358; LBC-A 1355; LBC-A 1347
Pipa carvalhoi	un	adult	Ilhés (BA)	bmcw	LBC-A 1426; LBC-A 1427; LBC-A 1428
Pristimantis paulodutrai	un	adult	Ilhéus (BA)	bmcw	no number
Rhinella icterica	un	adult	Vale do Paiol (MG)	pmc	LBC-A 1406; LBC-A 1386; LBC-A 1395
Rhinella jimi (from semiarid, currently R. diptycha)	un	adult	Angicos (RN)	pmc	LBC-A 892; LBC-A 893; LBC-A 1134
Rhinella schneideri (from savannah, currently R. diptycha)	un	adult	Ilhéus (BA)	pmc	LBC-A 1019; LBC-A 1098; LBC-A 1058
Siphonops annulatus	un	adult	Ilhéus (BA)	bmcw	LBC-A 1399 ; LBC-A 1400; LBC-A 1401
Trachycephalus mesophaeus	un	adult	Ilhéus (BA)	bmcw	LBC-A 1396; LBC-A 1029; LBC-A 1151

Notes. N = number of specimens; LBC-A = Amphibian Collection of the Laboratory of Structural Biology, Butantan Institute; pmc = parotoid manual compression; bmcw = body manual compression in water; un = unidentified sex.

*Secretion of Bufo bufo was obtained from Latoxan Laboratories S.A.S. (Portes lès Valence, France, product # ID L3101).
